# Some Discussions on Tuberculosis, Etc.

**Published:** 1890-01

**Authors:** 


### Selections.

### Some Discussions on Tuberculosis, etc.

The congress of physicians and naturalists recently held in Heidelberg, drew forth some interesting papers and discussions.

At the debate on tuberculosis in the Congress, Prof. Virchow opened the discussion by saying that Koch's bacillus did not explain all problems connected with tuberculosis, and that individual, hereditary and possibly also other, as yet unknown, factors, probably play a more significant *role* in this affection than is usually believed at present. The hereditary element, at least, is no longer to be underrated since the tubercle bacilli have been discovered in the genital organs of parents, the offsprings of whom were known to be consumptives. Dr. Sonnenschein, of Worms, called attention to the origin of constitutional diseases, and of consumption in particular, from milk. He believes that a great portion of infants and children, dying of tuberculosis, have been infected from the milk of diseased cows. It can not be doubted that the affection of cows, which the Germans call *Perlsucht*, is a tubercular process, and that, provided the udder itself is implicated, the milk from such cows is under certain circumstances capable of rendering man tuberculous. The fact that *Perlsucht* and consumption are identical affections ought to be published broadcast and the sale of milk from cows thus affected rigidly forbidden. It has besides been proven that the composition of cow's milk becomes deteriorated and the milk itself unfit for use if the animal is fed on certain waste products of factories (beet-sugar refineries and distilleries). Children taking such milk contract the most serious intestinal catarrhs, and perish in large numbers. It will therefore be the duty of the State authorities to rigidly superintend the feeding of so-called milk-cows, and that of physicians to enforce the use of milk in a state of sterilization and in sterilized vessels.

Prof. Victor Meyer, the successor of Bunsen, read a valuable paper on "Chemical Problems of the Present Time." The author alluded to the great progress in the science of chemistry, made by the labors of men like Bunsen, Hoffmann, Rekule, Van't Hoff, Baeyer, and Wisclicenus. Demetrius Mendeljeff has the credit of having established a natural system of elements, and of having first pointed out that the properties of elements are functions of the atomic weight. His researches have led him to believe that the number of elements in existence is one hundred, although only seventy are yet known with certainty. He separates the elements into two groups of seven elements each, and five groups of seventeen elements each. This makes ninety-nine elements in all, to which as the one-hundredths hydrogen is to be added. This divination of thirty elements, as yet undiscovered, resembles somewhat the predetermination of Neptune in our solar system. Mendeljeff's studies, particularly in regard to the corrected figures of atomic weight and their comparison with the so-called homologous orders, point with certainty to the compound nature of elements. This novel view becomes strengthened by the results of pyro-chemical researches. A new chemistry will arise as soon as we are enabled to experiment with bodies at a higher temperature than 1700° (Centigrade) the melting point of platinum used for crucibles. The author referred to the numerous trials of synthetical chemistry, and thought it likely that albuminous bodies—starch and sugar—would soon be prepared artificially by synthesis as the transformation of the wood-fibre into starch is the subject of assiduous trials and as the systematical increase of the albuminous components of plants has been proven—theoretically at least—by Hollriegel, it can be hoped that the science of chemistry will continue to materially enhance the prosperity and welfare of mankind.

A similar and equally interesting topic was that discussed by Prof. Hertz, of Bonn, viz.: the relations between light and electricity. Recent investigations have shown that electricity and light are closely allied to each other, and that the former, like the latter, is caused by an undulatory motion of ether. Faraday and Maxwell were first to make researches in this direction. The author himself claims the credit of having first demonstrated that light produces electrical phenomena, and that,

*vice versa*, electric waves produce light waves. The author has overcome the difficulty of finding a sufficiently delicate means of measuring both kinds of waves by means of a swinging conductor with interrupted wire. It such a conductor is placed in the focus of a very powerful concave mirror, the conductor is seen to emit sparks and even to act as an electric polarizer.

Prof. Puschmann, of Vienna, read a highly entertaining essay on the importance of the study of history, especially of the history of the medical sciences, for the medical profession. The neglect of medical history has been very dearly paid for. Thus plastic operations, known already in ancient times, became forgotten and were in 1792 declared impossible by the medical faculty of Paris, until at the beginning of this century they reached Europe again by the way of India. Similarly known in ancient times, then forgotten and ultimately rediscovered, were the ligation and torsion of arteries, flap amputations, and turning in delivery. The doctrine of the contagiousness of consumption, taught by Hippocrates, was forgotten, and our modern bacteriological schools had to rediscover it.

The treatment of consumption consisted in milk-cures, sea voyages, and the sojourn in Egypt—methods like those approved to-day. Auscultation of the chest was also practiced by Hippocrates. The crossing of nerve fibres in the brain was known to Artacus, who explained on this basis the paralysis on one side of the body in lesions of the opposite side of the brain. Pliny taught that in cures for obesity, nothing should be drunk during meals and but little after them—a principle included in the modern obesity treatment. Nearly all remedies of importance used to-day were known to the ancients, even the fat contained in the wool of sheep, in which Liebreich found lanolin. Galen compared sound to a wave and respiration to combustion. Even Darwin's doctrine of evolution was, in principle, taught by Aristotele. It will thus be seen that the study of the history of medicine is by no means a merely entertaining occupation, but one replete with practically available results.

